# The clinical and cost-effectiveness of a self-management intervention for patients with persistent depressive disorder and their partners/caregivers: study protocol of a multicenter pragmatic randomized controlled trial

**DOI:** 10.1186/s13063-021-05666-y

**Published:** 2021-10-23

**Authors:** Ericka C. Solis, Ingrid V. E. Carlier, Nic J. A. van der Wee, Albert M. van Hemert

**Affiliations:** Department of Psychiatry, Leiden University Medical Center (LUMC), Albinusdreef 2, 2333 ZA Leiden, The Netherlands

**Keywords:** Persistent depressive disorder, Chronic depression, Self-management, Psychiatric rehabilitation, Effectiveness, Partner/caregiver

## Abstract

**Background:**

After regular treatment, patients with persistent depressive disorder (PDD) may remain in specialized psychiatric outpatient care without achieving remission. Lacking other options, these patients often receive long-term, non-protocolized care as usual (CAU) that does not involve the partner/caregiver of the patient. Although the revised depression treatment guidelines suggest focusing on psychiatric rehabilitation and self-management as the next treatment step for PDD, an evidence-based cost-effective self-management protocol for PDD is lacking. This study investigates the “Patient and Partner Education Program for All Chronic Illnesses” (PPEP4All) as a brief self-management protocol that could lead to lower costs, higher quality of life, and less disease burden in PDD patients and their partners/caregivers.

**Methods:**

Presented is the rationale and methods of a multicenter pragmatic randomized controlled trial to evaluate the clinical efficacy and cost-effectiveness of PPEP4All for patients with PDD and their partners/caregivers. In accordance with current recommendations, a mixed methods research approach is used with both quantitative and qualitative data. A total of 178 eligible outpatients with PDD and their partners/caregivers are recruited and randomized to either PPEP4All or CAU. Those assigned to PPEP4All receive nine weekly self-management sessions with a trained PPEP4All therapist. Primary and secondary outcome measurements are at 0, 3, 6, and 12 months.

**Discussion:**

This project will result in the implementation of a self-management intervention for patients with PDD, meeting an urgent need in mental healthcare. Using PPEP4All can optimize the quality and efficiency of care for both patients with PDD and their partners/caregivers.

**Trial registration:**

Netherlands Trial Register Identifier NTR5973. Registered on 20 July 2016.

**Supplementary Information:**

The online version contains supplementary material available at 10.1186/s13063-021-05666-y.

## Background

Approximately 10–17% of persons with major depressive disorder (MDD) suffer a chronic course, or a continuous period of at least 2 years [1]. Two types of depressive disorders have a prolonged duration: chronic major depressive disorder and dysthymic disorder [2]. These were combined into one syndrome, persistent depressive disorder (PDD), in the latest Diagnostic and Statistical Manual of Mental Disorders (DSM-5) [3]. Compared to less persistent forms of depression, PDD has a 3–6% lifetime prevalence [3–7] and adverse consequences for patients, such as impaired functioning in social and family relationships, high disease burden for both patient and partner/caregiver, reduced performance in work, comorbid psychiatric and somatic problems, and an increased suicide risk [8, 9].

Many patients with PDD remain in specialized outpatient care without achieving remission, even after having been treated according to the international multidisciplinary depression guidelines [4, 10–12]. Clinicians are resigned to providing long-term supportive non-specific treatment (i.e., care as usual), due to the considerable suffering of patients and the absence of alternative treatment options [13]. More intensive prolonged care, however, often fails to improve therapeutic response; previous research has shown that patients with increasing depression symptom levels have a poor treatment prognosis despite receiving more intensive care [14–16]. The ongoing unsuccessful treatment leaves both patients and clinicians feeling powerless and frustrated [13]. Moreover, this treatment often disregards the partner/caregiver of the patient, although involvement of the partner/caregiver has been shown to be beneficial in improving treatment results of the patient and reducing the partner’s psychosocial burden concerning patient’s disease [17–21].

The revised multidisciplinary guidelines for depression treatment [11, 22–27] advise focusing on psychiatric rehabilitation as the next therapeutic step for PDD patients with inadequate treatment results. Psychiatric rehabilitation using self-management interventions focuses less on symptom recovery and more on restoring psychosocial functioning and enhancing patient autonomy. Individuals with PDD learn to set realistic goals (e.g., regarding work reintegration) and to cope by adjusting their activities to compensate for restrictions caused by the chronic illness. To date, there is still an urgent need in specialized mental healthcare for a brief evidence-based, cost-effective treatment protocol concerning self-management for treatment-resisting persons with PDD that also involves the partner/caregiver and replaces long-term non-specific usual care.

As a possible solution, we propose the use of the self-management program named the “*Patient and Partner Education Program for All Chronic Illnesses*” (PPEP4All). This program was originally named the “*Patient and Partner Education Program*” (PPEP) and developed for homogenous groups of patients with chronic somatic diseases, like Parkinson’s disease and Huntington’s disease, and tested by EduPark, a research consortium with seven participating European countries [18, 28–30]. Later, PPEP was tested in a heterogeneous group of patients with various chronic somatic disorders and comorbid long-term depression and/or anxiety, with positive results, after which the program name was changed to PPEP4All [18]. The structure and substance of the eight sessions are the same; however, the wording of the program and workbooks was altered for inclusivity of various diseases [31–33], and the present PPEP4All program includes one additional session regarding coping with chronic depression (i.e., “Suicide prevention & relapse plan”).

Research has already shown that PPEP is effective: patients with Parkinson’s disease showed a significant improvement in quality of life after completion of PPEP [19]. Moreover, patients with symptom-present Huntington’s disease showed a significant improvement in active coping and seeking social support and reported fewer behavioral problems and less anxiety. After 6 months, they also showed an improvement in psychosocial burden related to their chronic illness [34]. Patients with chronic pituitary disorders showed significant higher self-efficacy after following PPEP [35]. Moreover, after following PPEP4All, patients with various chronic somatic disorders and comorbid chronic depression showed an improvement in depression scores [18]; an indication that PPEP4All could also be applied to mental healthcare, which was the starting point for the present study. The cost-effectiveness of PPEP/PPEP4All has not yet been investigated.

The present study evaluates the clinical- and cost-effectiveness of PPEP4All in patients with PDD and their partners/caregivers. Additionally, this project aims to further our understanding of the healthcare needs for patients with PDD and elicit direct feedback about PPEP4All from patients, partners/caregivers, and clinicians via qualitative interviews. Compared to usual care for PDD, we expect that PPEP4All will lead to an improved quality of life, fewer psychiatric symptoms, and more mental resilience in patients (using superiority testing). Second, we expect that PPEP4All will lead to lower psychosocial burden from chronic depression for both patients and their partner/caregiver. Third, due to the briefness and anticipated higher effectiveness of PPEP4All, we expect lower societal and healthcare costs, compared to care as usual.

## Methods

### Study design

In this multicenter, pragmatic randomized controlled trial (RCT), the protocolized PPEP4All self-management intervention is evaluated against care as usual (CAU) in specialized outpatient mental health care clinics in the Netherlands. Measurements are at 0 (pre-treatment), 3 (post-treatment), 6 (first follow-up), and 12 months (second follow-up), with assessors/data-analysts blind to randomization status. In accordance with current recommendations (in the context of RCTs) [36–41], we use a mixed methods research approach with both quantitative and qualitative data, where the qualitative study is nested in the main RCT project. The qualitative data complements the quantitative data and can facilitate the practical implementation of the intervention, strengthen the clinical relevance of our results, and optimize the PPEP4All intervention by adding the perspectives of patients, partners/caregivers, and clinicians. Moreover, the study protocol conforms to the Standard Protocol Items: Recommendations for Interventional Trials (SPIRIT) guidelines (see Additional file 1).

### Setting and participants

Eligible patients are recruited from five Dutch mental healthcare organizations, with 11 individual locations, that offer outpatient treatment for depressive disorders (see Acknowledgements). The primary/lead research center of the study is the Department of Psychiatry of the Leiden University Medical Center (LUMC). The inclusion criteria for participating patients are as follows: (1) diagnosis of recurrent or chronic major depressive disorder (duration of at least 2 years) according to the DSM-IV [2] or PDD according to the DSM-5 (non-severe comorbidity is allowed, e.g., anxiety disorder) [3]; (2) age of 18 years or older; (3) treatment indication for rehabilitation as specified by the clinician, with a minimum of at least one previous psychological treatment and at least two medication trials, with unsatisfactory results, according to the international multidisciplinary guidelines for depressive disorders [11, 22–27]. Patients with recurrent or chronic depression who do not wish to complete all treatment steps of the depression guideline (e.g., refuse medication) can also enroll in the study. Patients with bipolar disorder type II may also participate considering they generally spend much more time in a depressed state than in a hypomanic state, making it difficult to differentiate from recurrent depression [42]. Exclusion criteria for patients are as follows: severe psychopathology (e.g., schizophrenia, current psychotic state, severe substance addiction, bipolar disorder type I); acute and severe risk of suicide; severe somatic disorders that are too disabling or render the patient immobile; severe cognitive problems such as dementia; expected dosage adjustments of medication during PPEP4All; and insufficient fluency in the Dutch language.

The inclusion criteria for partner/caregiver to participate in the study and PPEP4All program for partners/caregivers are as follows: (1) ability to participate in at least three sessions of the intervention, (2) not currently receiving active psychotherapy, and (3) agreement from the patient for the partner/caregiver to participate.

### Recruitment, enrollment, and allocation

Psychiatric nurses and psychiatrists of the participating mental healthcare locations identify patients eligible for participation according to the above inclusion/exclusion criteria, and they introduce research participation and provide information letters. Patients who are interested and consent to participate in the study sign an informed consent form, which is then sent to the primary research center (LUMC). On this informed consent form, participants also indicate whether they want to be invited to a follow-up nested qualitative interview study in which we evaluate their satisfaction with PPEP4All and their process of implementing coping strategies for chronic depression (see section “Qualitative data”).

To check the inclusion and exclusion criteria formally, a trained research assistant checks the DSM-IV depressive disorders with the Dutch translation of the Mini-International Neuropsychiatric Interview (MINI interview, modules A, B, and C regarding depression, dysthymia, and suicidality). The MINI interview is a well-validated diagnostic interview used to identify psychiatric disorders, with excellent interrater and test-retest reliability [43, 44].

Allocation of participants is performed by an independent data coordinator/data manager, who is not involved in the data collection/analysis (GC; see Acknowledgements). Participants are allocated to either PPEP4All or CAU, using a stratified randomization schedule (stratified by gender and research center) designed by an independent statistician of the Department of Medical Statistics and Bioinformatics of the LUMC.

**Fig. 1:**
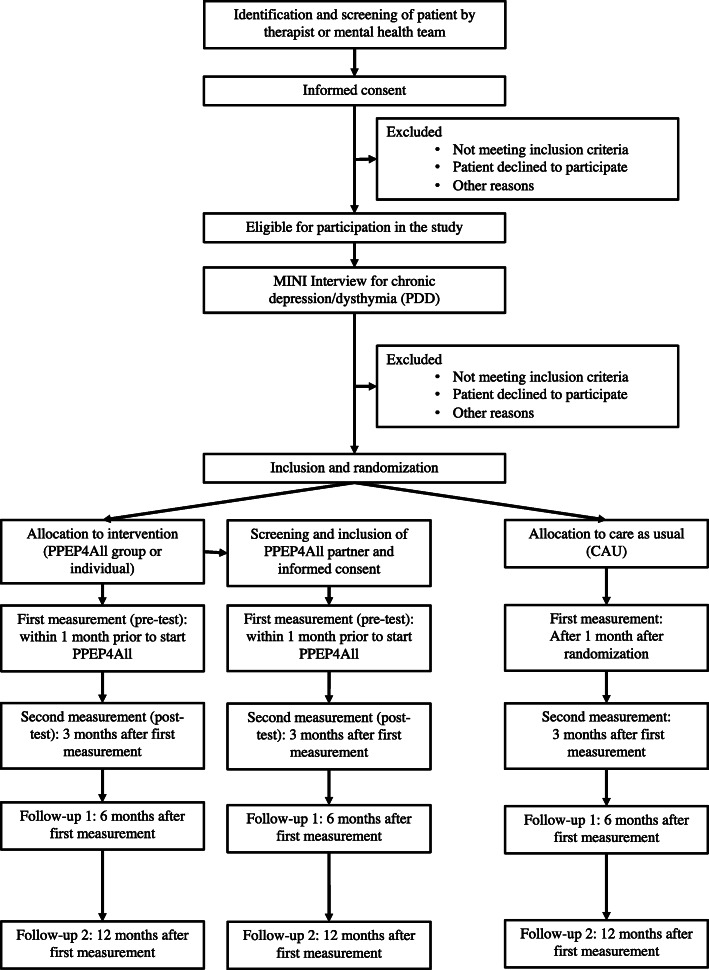
Study design and flow. *Notes:* MINI = Mini-International Neuropsychiatric Interview. PDD = Persistent depressive disorder. PPEP4All = Patient and Partner Education Program for All Chronic Illnesses. CAU = care as usual

For the patients allocated to PPEP4All, their main caregiver (e.g., partner, close family member, or close friend) is invited to participate in the project. The data manager makes an appointment with the partner/caregiver, and they sign an informed consent before the first measurement. All participants are informed that participation is voluntary and that they can withdraw from the study at any time without consequences. Participants receive a 20-euro gift card to thank them for their efforts. An overview of the study design and patient flow is provided in Fig. 1.

### Assessment and instruments

**Fig. 2:**
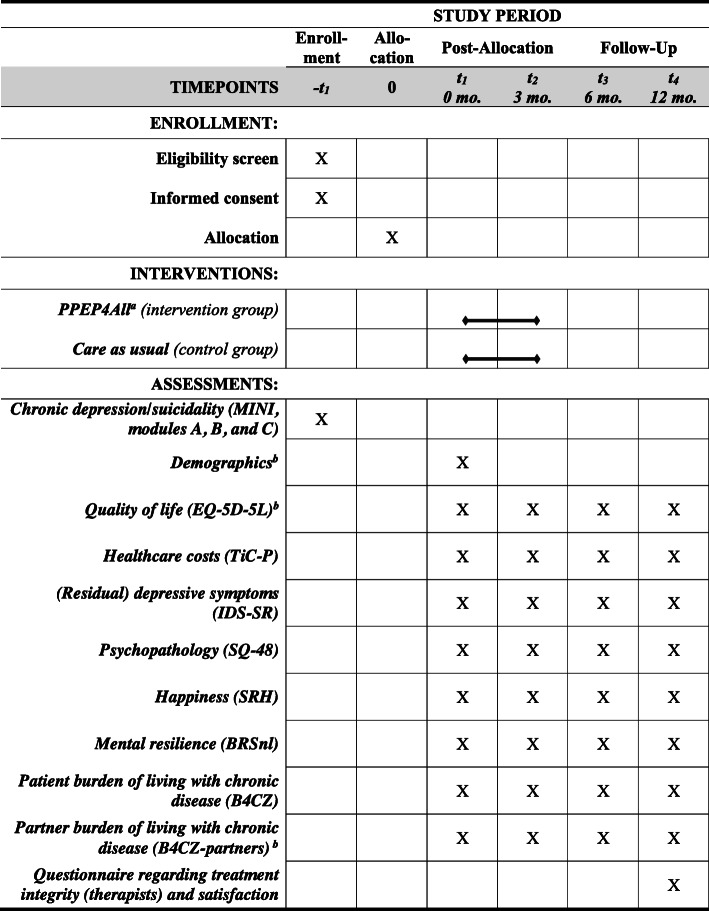
Standard Protocol Items: Recommendations for Interventional Trials (SPIRIT) schedule of enrollment, interventions, and assessment of the study. Notes: MINI = Mini-International Neuropsychiatric Interview [35, 36]. EQ-5D-5L = EuroQoL-5 with visual analogue scale [40–44]. TiC-P = Trimbos Medical Technology Assessment questionnaire for psychiatric illness-associated costs [45]. IDS-SR = Inventory of Depressive Symptomatology, self-rated [46–48]. SQ-48 = Symptom Questionnaire-48 [49–52]. SRH = self-rated happiness [53–55]. BRSnl = Brief Resilience Scale [56, 57]. B4CZ = Questionnaire on Burden of Chronic Disease for Patients (or Partners) [58, 59]. ^a^The PPEP4All intervention is mainly provided as a group treatment but can also be offered as an individual treatment. ^b^These questionnaires are administered to partners/caregivers in the study

As mentioned, the measurement timepoints are at 0, 3, 6, and 12 months; the latter two are follow-up measurements. The first measurement is completed within 1 month prior to the start of the treatment. The study is conducted using a Routine Outcome Monitoring (ROM) system, which periodically measures the presence and severity of psychiatric symptoms in patients with a battery of psychometric instruments and thus monitors therapeutic progress [45–47]. To assist in the administration of the ROM measurements, a web-based application called QuestManager is available. At each timepoint, the questionnaires are completed online via QuestManager (see Fig. 2 for the study schedule and overview of the questionnaires). If preferred or necessary (e.g., computer problems), questionnaires may be completed on paper. The participant may choose to complete the questionnaires at the location of their choice: at their home, the mental health clinic, the research center, or by telephone.

At the first measurement (i.e., 0 months), participant demographics are registered, and the primary and secondary outcome questionnaires are completed. At the second, third, and fourth measurements (i.e., 3, 6, and 12 months), the outcome questionnaires are again completed.

The *primary outcome measures* pertain to quality of life and healthcare costs (see Fig. 2). Quality of life is measured for both patients and partners/caregivers using the EuroQoL-5 (EQ-5D-5L, including the visual analogue scale, 5 items) [48–52]. Using the EQ-5D-5L, we calculate the mean quality-adjusted life years (QALYs) gained, which is used in the cost-effectiveness analysis (CEA; see “Economic evaluation”). Healthcare consumption (such as contact with healthcare professionals, medical costs, and productivity loss) are measured with the Trimbos Medical Technology Assessment questionnaire for psychiatric illness-associated costs (TiC-P; 30 items) [53].

The *secondary outcome measures* are shown in Fig. 2. Symptoms, psychopathology, and well-being, for patients only, are measured using the Inventory of Depressive Symptomatology, Self-Report (IDS-SR; 30 items) [54–56]; the Symptom Questionnaire-48 (SQ-48; 48 items) [57–60]; and the Self-Rated Happiness survey (SRH; 1 item) [61–63], respectively. In addition, mental resilience of patients is measured using the Brief Resilience Scale (BRSnl; 6 items) [64, 65]. The burden of chronic disease for both the patient and partner/caregiver is measured by the Questionnaire on Burden of Chronic Disease for Patients (B4CZ; 20 items), and the Questionnaire on Burden of Chronic Disease for Partners (B4CZ partners; 16 items), respectively [66, 67]. Directly after the final measurement, patients complete an additional evaluation survey pertaining to the following: (1) treatment modality (group or individual treatment), (2) treatment satisfaction, (3) number of therapy sessions, (4) medication changes (yes/no); and (5) PPEP4All treatment adherence.

## Interventions

### Self-management intervention (PPEP4All)

**Fig. 3:**
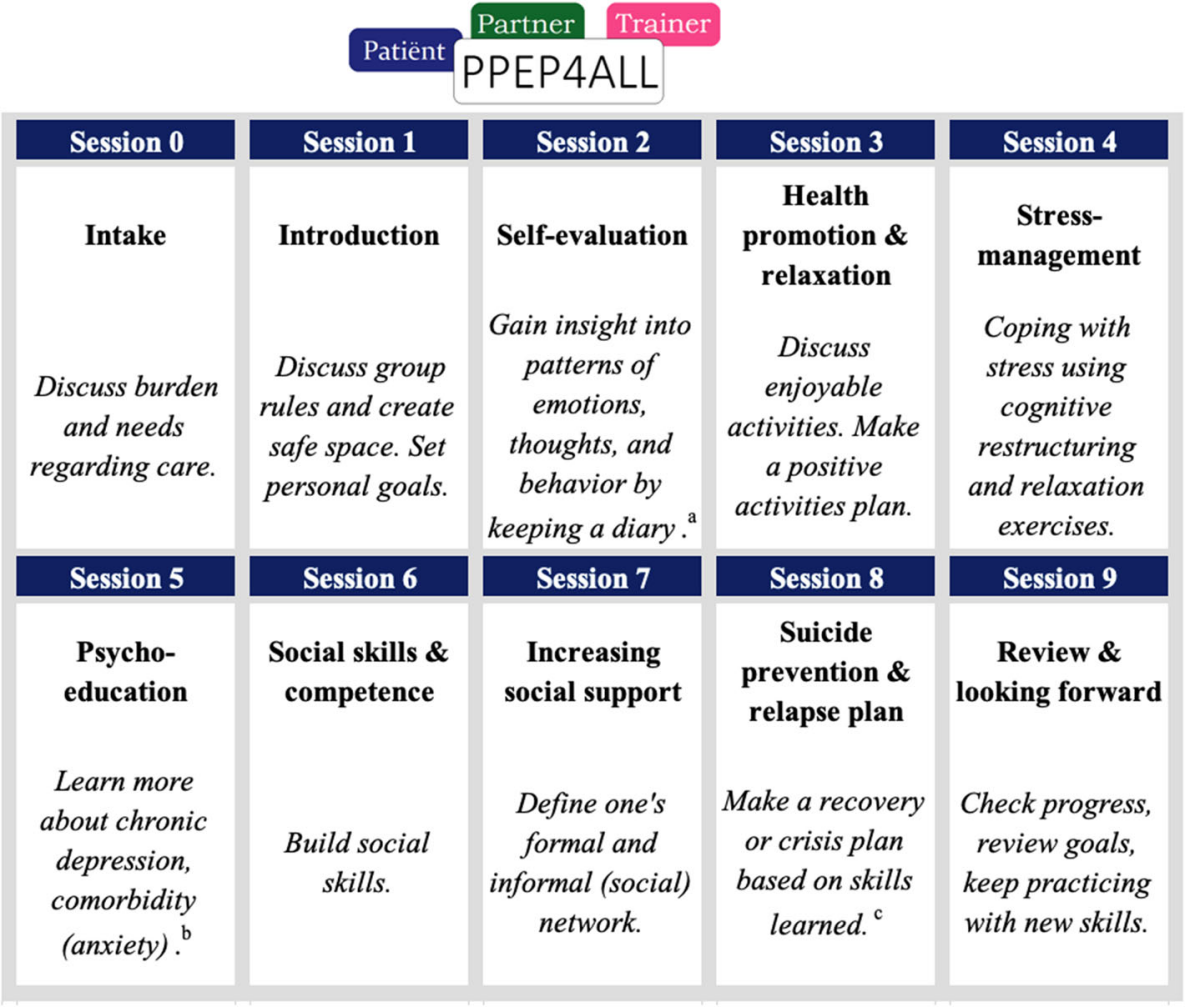
An overview of the self-management themes of the PPEP4All sessions for the patient and the partner/caregiver. *Notes:* PPEP4All = Patient and Partner Education Program for All Chronic Illnesses. Patient and partner/caregiver programs include the same themes with the exception of sessions 5 and 8. Each session has a general structure of discussing previous homework, presenting active information (i.e., the theme), performing an active exercise, then providing homework and a preview of the next session. The trainer is the PPEP4All therapist. ^a^Participants also gain insight by using a body awareness exercise. ^b^In partner/caregiver program, session 5 focuses on caregiver burden and stress (overload) and relevant challenges. ^c^In partner/caregiver program, session 8 focuses on making a self-care plan

Each of the nine PPEP4All sessions focuses on specific self-management themes, such as psychoeducation about chronic depression, stress-management using cognitive restructuring and relaxation techniques, reactivation with positive activity planning, social skills building, and mobilization of one’s social network. Although the original PPEP4All program had eight sessions, it has been suggested that an extra PPEP4All session may help sustain enhanced quality of life [19]. PPEP4All in this project includes a ninth session focusing on suicidality, dealing with crises, and relapse prevention. Regarding the latter, participants make a recovery or self-care action plan together with the PPEP4All therapist, using the skills and information learned during the program. For an overview of the PPEP4All self-management themes, see Fig. 3.

The partner/caregiver program (separate partner group) is the same as the patient program with only one difference: session five focuses on dealing with caregiver burden and patient suicidality (see Fig. 3). The patient and partner/caregiver receive PPEP4All workbooks with homework assignments after each session to practice skills and integrate information. Although the original protocol describes a minimum of 5 and a maximum of 10 participants per group [17], we allowed groups to start with as few as 3–4 participants to avoid undue waiting times.

The PPEP4All program is eclectic, including theoretical influences of system theory (“patient system”) [68, 69], cognitive behavioral theory (“(dys) functional cognitions”) [70], social cognitive theory (“self-efficacy”) [71], stress-coping model (“coping strategies”) [72, 73], transtheoretical model (“motivation to change”) [74]; bio-psycho-social model (quality of life) [75–77], and generic model of self-management [78].

At the time of this project, there are other self-management programs available, such as the *Wellness Recovery Action Plan* (WRAP; a self-designed prevention and wellness process, mainly used for severe mental illness) [79, 80]; *Illness Management and Recovery* (IMR; a standardized psychosocial intervention for severe mental illness like schizophrenia) [81]; and *Self-management for Chronic Anxiety and Depression* (SemCAD; for chronic depressive and anxiety disorders) [82–84]. PPEP4All, however, has advantages when compared to these other interventions. First, PPEP4All involves and engages the partner/caregiver in the treatment process, which has been shown to be effective in reducing the partners’ psychosocial burden concerning the patients’ illness as well as the patients’ outcome [17–21]. A key feature of PPEP4All is that the patient and partner/caregiver receive separate sessions. Maintaining separate sessions allows both patient and partner/caregiver to speak freely about their personal situations. Second, the program can be offered as a group or individual intervention. The treatment modality is determined by the patient and his or her therapist in a process of shared decision making. Third, patients with depression often have comorbid psychiatric or somatic disorders [14, 85]; the PPEP4All program provides a general toolkit with which all these issues can be addressed. Fourth, compared to the other self-management programs, each session of PPEP4All is highly structured, with both patients and partner/caregivers working according to their PPEP4All workbook.

The clinicians of the participating mental health clinics who administer the program are called PPEP4All therapists. Each therapist receives a treatment manual and completes a 3-day certified course with the PPEP4All founder [17, 18, 31, 32, 86, 87]. The training combines homework/self-study with lectures, assignments, group discussions, role play, and self-assessment. The 34 PPEP4All therapists in this project are mainly psychiatric nurses or psychiatric nurse specialists and three are psychologists. After completing their PPEP4All training, all PPEP4All therapists in this study could receive continuous support with regular booster sessions, intervision, consultation with the PPEP4All founder, and an annual national PPEP4ALL symposium.

### Care as usual (CAU)

To compare PPEP4All with what is currently being offered in mental health clinics, the participants randomized to the control group receive care as usual (CAU), thus increasing the ecological validity of the present study. This standard treatment for patients with PDD concerns mostly long-term, non-protocolized supportive care from a psychiatric nurse, with pharmacological maintenance therapy from a psychiatrist. CAU mostly has an individual treatment modality and does not include the partner/caregiver of the patient. Patients allocated to CAU in this study generally continue the standard treatment with their own therapist.

## Treatment integrity

To evaluate the treatment integrity of PPEP4All, each PPEP4All therapist completes a therapy protocol checklist at the end of each group or individual session. On this protocol checklist, the PPEP4All therapist indicates whether and how the provided treatment deviated from the treatment protocol (e.g., the themes mentioned in Fig. 3). It is not possible to record therapy sessions with a video or audio recorder in participating mental health clinics due to privacy law and regulations. Morever, participant attendance is recorded on a separate attendance list and provided to the researchers by the PPEP4All therapist. For CAU, without a specific treatment protocol, we cannot assess its treatment integrity. However, after the final fourth measurement, we collect information about treatment type and modality within CAU, including treatment satisfaction and general medication changes during the course of the research project.

## Sample size and power calculation

We based our sample size on the effect size of previously conducted randomized controlled trials that evaluated relevant interventions in a similar setting in patients with chronic or treatment-resistant depression and that reported a quality of life outcome measure [83, 84, 88–90]. We can expect an effect size around 0.35 [90]. Considering a power level of 80%, alpha of 95%, and equal allocation ratio, we would require 81 participants per group [91–95]. With an additional 16 persons to account for an expected attrition of 10% [96], we expect to include a total sample of 178 participants (2 × 89).

## Data analyses

In this project, we examine both quantitative and qualitative data.

### Quantitative data

Using the data collected from the questionnaires, we examine the clinical and economic effectiveness of PPEP4All (in collaboration with the LUMC Department of Medical Decision Making). This is further specified below.

### Clinical effectiveness

The mean quality-adjusted life years (QALYs) are estimated using EQ-5D-5L data from four assessments. For the *primary data analysis*, we perform an analysis of covariance for the QALY’s using the post-test EQ-5D-5L results with the pre-test scores as covariate [92–94]. In this way, we test whether there is a difference between PPEP4All and CAU, accounting for the variation where the patients started at the first measurement. For the *secondary data analyses*, secondary parameters are tested using weighted generalized estimating equation (GEE) analyses [97, 98]. Clinical effectiveness analyses are examined using superiority testing.

Moreover, we examine tailored care by further investigating for which groups PPEP4All is the most effective. If possible, subgroup analyses are performed for age, gender, ethnicity, and intervention modality (group/individual). In this context, we explore relevant socio-demographics (e.g., education, living situation, and work) and clinical variables (e.g., comorbidity, baseline severity of disease).

For all outcome analyses, an intention-to-treat (ITT) approach is used, according to the CONSORT statement [99]. Analyses are conducted in SPSS, Stata or R.

### Economic evaluation

We perform two economic evaluation analyses (see below). The first is a cost-effectiveness analysis (CEA) to determine whether PPEP4All (compared to CAU) is favorably cost-effective at 1-year follow-up. The second is a budget impact analysis (BIA). The economic evaluation is based on the TiC-P questionnaire (see Fig. 2). The TiC-P questionnaire assesses healthcare utilization such as general practitioner (GP) visits and non-healthcare use such as absence from work, reduced efficiency at work, difficulties with job performance (absenteeism from paid work), and production losses without absenteeism from paid work (e.g., presenteeism). Using the area-under-the-curve method for the utility scores results in QALY outcome per patient.

#### Cost-effectiveness analysis (CEA)

Direct costs per patient in PPEP4All versus the costs per patient in CAU are compared. The differences in mean costs and effects between strategies are compared with two-sided bootstrapping. In a net-benefit analysis, costs are related to patient-reported outcomes and presented in a cost-effectiveness acceptability curve (CEAC), which indicates the probability of PPEP4All being cost-effective. No discounting is applied considering the time horizon of 1 year. This economic evaluation is approached from a societal perspective and includes costs due to healthcare resource utilization (i.e., healthcare costs) and costs attributable to production losses [100]. Sensitivity analysis will test whether estimates are sensitive to plausible changes in perspectives (societal or healthcare perspective) and indirect costs (friction cost method and human capital approach). Furthermore, in an additional analysis, we extrapolate cost and effect estimates to a lifetime time horizon, in accordance with the recent Dutch directive for conducting economic evaluations in healthcare [101, 102]. In the base-case analysis, costs of absenteeism from paid work are calculated using the friction cost method, which estimates the indirect costs of disease, mainly occurring during the time it takes to replace a worker [103–106].

In this project, costs are calculated using standard unit prices published in the most recent Dutch Costing Manual [101, 102]. If references are not available, costs are estimated by cost research experts (i.e., LUMC Department of Medical Decision Making). The costs are divided into healthcare costs, costs of patients and partner/caregiver, and costs in other sectors (i.e., non-healthcare costs such as productivity costs). Healthcare costs include the costs of PPEP4All and other healthcare use during the first year of follow-up (e.g., GP visits, outpatient visits, hospital days, medication, home care, informal care). Costs of PPEP4All are based on micro-costing including time of caregivers and materials used. Costs of patients and partners/caregivers consist of time lost or productivity loss from paid/unpaid work of the patient, and time required for the PPEP4All meetings by partners/care givers.

#### Budget impact analysis (BIA)

In addition to the CEA, a BIA is performed according to the International Society for Pharmacoeconomics and Outcomes Research (ISPOR) Task Force principles [107, 108]. The BIA estimates the financial impact of implementation (i.e., adoption and diffusion) of PPEP4All in patients with PDD at the national level. The analysis is based on the costs estimated during the study and the expected number of patients eligible for this treatment in the Netherlands. Costs of the treatment and other consequences of it will be included in the BIA. The BIA is conducted from the perspective of the different healthcare payers (public purse or budgetary framework [in Dutch: Budgetair Kader Zorg (BKZ)]; health insurers; health care providers) and from the societal perspective (i.e., including productivity costs). If the PPEP4All intervention appears to be cost-effective in patients with PDD, the following scenarios will be compared: (a) intervention not yet implemented, (b) intervention implemented in 100% of the target group, (c) intervention gradually introduced over a period of 3 years. Sensitivity analysis is performed on the costs of the intervention and the diffusion rate. Factors that determine the budget impact are determined. Costs are estimated per budget period (1 year) for a time horizon of 3 years. In the societal perspective and healthcare perspective, standard prices will be used [101]. For the BKZ and health insurer’s perspective, tariffs established by the Dutch Healthcare Authority (Nederlandse Zorgautoriteit (NZA)) are used.

### Qualitative data

All patients and partners/caregivers who give consent to be approached for the nested follow-up qualitative study are invited by telephone to participate. The participant may choose where the in-depth semi-structured interview is planned: at the participant’s home, at the main research location (LUMC), or over telephone. Participants are compensated for travel and given a 20-euro gift card to thank them for their time. Participants are allowed to withdraw at any moment of the qualitative study.

For each interview, we use topic lists, or interview guides (see Supplementary Topic Lists in Additional Files 2 and 3), which were evaluated in a pilot study of patients with PDD and revised as necessary [109, 110]. The topics and questions are presented to the participant as they naturally arise during the interview, to maintain the flow of the interview. The qualitative study includes two parts that examined: (a) use of coping strategies of PDD patients and their partners/caregivers for chronic depression/suicidality and their specific needs for care; (b) satisfaction of patients, partners/caregivers, and therapists with PPEP4All (or CAU). The two parts are discussed in further detail below (see “Coping with chronic depression” and “Satisfaction”).

The interviews are recorded using a digital audio recorder and then transcribed by research assistants using the dictation feature of the online Transcribe application (https://transcribe.wreally.com). This application does not store documents; audio files are deleted after the window is closed, making it a secure method that maintains confidentiality after transcription work. Personal data that can be traced back to the participant (e.g., names of participant, family members, or employer) are removed, and transcripts are saved under a participant code. The verified transcripts are then checked and analyzed by the researcher (ES) and a research assistant. Each interview and the resulting themes are discussed with the research assistant and tracked using an audit trail, to provide systematic and detailed documentation of analytical decision making during the process. Memo writing is utilized throughout the analytic process. Discrepancies in coding are discussed until consensus on the data is established. Because we used a qualitative emergent approach focused on gaining new insights, rather than developing a theory, our analytical approach in this study can be described as using “Grounded Theory lite” [111–116]. For the analysis, using Atlas.ti version 9, transcripts are first coded line-by-line, then coded axially into emergent conceptual categories [113, 116, 117]. We also use constant comparison, a method in which we continuously compare the concepts or themes between data and emergent coded categories and the data of the interviews [117, 118]. The main goal of the study is greater conceptual clarity and to build a conceptual framework of the experiences of participants with chronic depression regarding their treatment needs and use of self-management to meet these needs [119].

### Coping with chronic depression

First, we aim to answer the following research questions: *how do individuals with chronic depression manage their chronic condition, and what are their experiences in relation to potential facilitators and barriers, personal strengths and hindrances, and use of self-management coping strategies?* (see Supplementary Topic List A, or Additional File 2, which is partly based on a previous relevant topic list [110]). For this part of the qualitative study, we predetermined the number of required participants to ensure a sufficient sample. According to guidelines, we aim to include between 25 and 40 patients from the PPEP4All or CAU groups and 15 PPEP4All partners/caregivers to reach saturation of the themes [120–122].

### Satisfaction

Second, we aim to evaluate satisfaction with PPEP4All and investigate how we can optimize it to meet the needs of the patients with PDD and their partners/caregivers. Our approach is multi-faceted: we conduct in-depth interviews with patients, partners/caregivers, and PPEP4All therapists (see Supplementary Topic List B, or Additional File 3, which is partly based on the Mental Healthcare Thermometer [109]), and we collect data using an evaluation questionnaire at the end of the fourth quantitative measurement to elucidate the positive and negative aspects of PPEP4All or CAU. During the interview, participants may provide feedback and suggestions regarding PPEP4All, and they rate the PPEP4All program on a scale from 1 to 10. We will provide the average rating for each group. This satisfaction rating reflects an indirect measurement of the effectiveness of PPEP4All. For this part of the qualitative study, we aim to include between 25 PPEP4All patients, 15 PPEP4All partners/caregivers, and 10 PPEP4All therapists [120–122]. In addition, we strive to measure satisfaction of participants of CAU. The procedure for evaluating CAU is the same as PPEP4All: we use an evaluation questionnaire and a qualitative interview. During the interview, participants can rate CAU on a scale from 1 to 10 and provide additional information regarding their experience with CAU, such as suggestions regarding what kind of treatment techniques they would prefer as CAU.

## Ethics, data management, and dissemination

The study protocol, informed consent forms, participant education, and recruitment materials were approved by the Medical Ethics Committee (MEC) of the LUMC. If there are any subsequent modifications to the protocol which may impact the conduct of the study, including changes of study objectives, patient population, sample sizes, study procedures, or significant administrative aspects, these will be submitted as an amendment and reviewed by the MEC of the LUMC for approval. The approved protocol modifications will be communicated to research locations and participants by email or newsletter. Subsequent to initial review and approval, the MEC will review the protocol annually and the researcher will make safety and progress reports to the MEC annually and within 3 months of study termination. These reports will include the total number of participants enrolled and any particularities that should be reported to the MEC. Due to the low risk associated with study participation, annual audit of the study, data monitoring committee, and interim analyses are not required. Additionally, a data safety monitoring board is not required because we do not investigate a medical product and we do not foresee any major risks associated with study participation or with participation in the intervention provided by trained therapists.

Written informed consent will be obtained from all participants prior to the baseline assessment. All informed consent forms will be arranged and co-signed by the researcher or trained research assistant who provided information regarding the study. For the nested qualitative study, each participant gave written consent to be approached for participation in this additional study. Participants thereafter gave verbal consent to the study including consent to audio recording and transcribe their interviews.

In general, we followed the guidelines for Good Research Practice (GRP) [123–126]. To protect the confidentiality of data and privacy of patients, we use a unique research code for each participant on all research-related participant forms, such as contact forms or paper questionnaires. This enables the research team to identify individuals without using names. The list linking the participant code to personal information is kept in a secure electronic database with access limited to the designated researcher (ES) and independent data coordinator (GC). The list may be assessed and the main mental health clinician of the patient informed, if required for the sake of the safety of the participant. All electronic research-related participant information is stored on a protected network in a secure file with limited access. The hardcopy paper research information is kept in a locked cabinet in a secure area of the LUMC department. Any documents with information traceable to the participant are kept in locked files separate from the patient data with limited access. To uphold high standards of research, all researchers, research coordinators, and research assistants successfully completed the research certification for Good Research Practice (GRP). Additionally, all research team members completed a training workshop regarding the trial procedures and questionnaires, use of QuestManager, and administration of the MINI screening interview.

Moreover, after the data analysis phase, patient privacy will be further maintained. The results obtained from the questionnaires will be described on a group level to prevent the data being traced back to a single person. Data will be kept on the secure network for maximum 3 months at the end of the research project as the embargo period, for the sole purpose of completing data analysis, as per the policy of the funding sponsor. Once the final report of the study is available, the study results will be extensively disseminated to the international scientific community in the form of peer-reviewed journal articles, giving preference to open-access journals.

After the embargo period, the pseudonymized data will be stored for 15 years on an online Dutch meta-data catalogue called the Data Archiving and Networked Services (DANS, www.dans.knaw.nl) together with a data dictionary, as is common practice for mental healthcare research in the Netherlands. Only the designated researchers will have access to the final data set. External researchers may get access to the final trial dataset from the designated team on reasonable request.

## Discussion

### Scientific and clinical importance of this study

The present study examines the clinical- and cost-effectiveness of a brief self-management intervention, namely PPEP4All, in patients with PDD and their partners/caregivers. Although self-management is more common in the treatment of somatic illnesses, it is gaining popularity in mental healthcare. Psychiatric rehabilitation via self-management is the next step in the revised international multidisciplinary treatment guidelines for chronic depression after psychotherapy and pharmacotherapy have been attempted with inadequate results. In this context, there is an urgent need in mental healthcare for a brief specific self-management protocol that could replace the long-term non-specific care for patients with PDD (care as usual).

### Study implications

The findings of this study will have potential implications for the implementation of self-management on a national level in the Netherlands and could be further incorporated into the multidisciplinary treatment guidelines for depression. The Netherlands has recently seen a shift in the mental healthcare system due to tighter healthcare budgets. Mental healthcare clinics are looking for empirically supported protocolized treatments that can reduce healthcare costs. With this in mind, PPEP4All is a brief self-management intervention that can be provided when depression treatment leads to unsatisfactory reduction of symptoms, typically after a 2-year treatment period. The expectation is that empowering patients via PPEP4All will decrease the number of ongoing supportive sessions of CAU, for which there is currently little to no empirical evidence [13].

The present study has several strengths. First, in line with the pragmatic nature of the project, we focus on external validity: we evaluate the standard care (CAU) already provided by healthcare professionals in multiple mental healthcare clinics. Moreover, the broad inclusion criteria allow the recruitment of patients with a wide range of comorbidity, as typically seen among persons with chronic or recurrent depression. We expect the resulting sample to be broadly representative of outpatients and generalizable to the population of persons with chronic depression. We selected only the most crucial research questionnaires to limit the burden on the patients; therefore, we expect limited effect of the test battery on patient drop-out. Additionally, by using a mixed methods research approach, we are able to evaluate both quantitative and qualitative aspects of PPEP4All from multiple perspectives (patient, partner/caregiver, clinician).

### Challenges concerning trial recruitment and implementation

We wish to report some early challenges we faced in the recruitment of participants and implementation of our study design and PPEP4All in the participating research centers. After all, patient recruitment is a key determinant of success or failure for clinical trials [127–129].

Firstly, we learned that recruiting only patients with a primary diagnosis of chronic depression/PDD limited our ability to also recruit potential participants with secondary, or comorbid, PDD (e.g., bipolar disorder type II participants with comorbid PDD). After careful deliberation, we allowed these participants to enroll in our study considering that PPEP4All is a generic self-management program that could potentially be used for a wide range of psychiatric disorders and its focus is on recovery, stabilization, and reintegration into the community [130].

Secondly, we had to make the following adjustments to reduce participant drop-out [93]. Initially, we had proposed providing PPEP4All as group treatment, because a group could be the most cost-effective and efficient modality for mental healthcare. However, several patients with PDD preferred individual PPEP4All and therefore refused to participate. This possible preference for individual PPEP4All was also reported to us by the Client Council of the participating centers. For this reason, although PPEP4All is still primarily offered as a group treatment, we also allow the option to provide PPEP4All as an individual treatment (same protocol). In addition, for patients with a preference for group PPEP4All, we lowered the minimum threshold of required group participants to three rather than the original five, which allowed us to start PPEP4All groups without a long waiting period. Moreover, some patients who explicitly wanted PPEP4All withdrew their study participation after they were randomized to CAU. We remedied this with the message that, if necessary, these patients could receive PPEP4All after CAU and completing at least their first or second follow-up.

## Conclusion

This self-management intervention, PPEP4All, could fill an urgent need in mental healthcare and provide further support for the use of psychiatric rehabilitation via self-management for patients with PDD and their partner/caregiver. Using psychiatric rehabilitation as the next step in the treatment guideline for PDD without remission could replace the non-protocolized usual care and optimize the quality and efficiency of depression care. PPEP4All is expected to reduce costs for the mental health organizations and empower patients to achieve a higher quality of life.

## Trial status

This trial is currently recruiting patients, which started on 1 April 2017 and is anticipated to end on 1 April 2021. The trial is expected to end on 31 December 2022. The protocol is Version 7 dated 1 February 2021.

## Supplementary Information

**Additional file 1.**

**Additional file 2.**

**Additional file 3.**

## Data Availability

Datasets generated and/or analyzed during the current study will be pseudonymized and stored on an online Dutch meta-data catalogue called the Data Archiving and networked Services (DANS, www.dans.knaw.nl), according to the funding sponsor policy, with access limited to a designated team within the Department of Psychiatry of the Leiden University Medical Center. External researchers may get access to the final trial dataset from the designated team on reasonable request. The (intellectual) property rights with regard to the generated data will reside at the Leiden University Medical Center. Anonymized results will be published in peer-reviewed journals and presented in international conferences.

## Abbreviations

B4CZ: Questionnaire on Burden of Chronic Disease, version for Patients or Partners
BIA: Budget impact analysis
BRSnl: Brief Resilience Scale-Dutch translation, 6 items
CAU: Care as usual
CEA: Cost-effectiveness analysis
CEAC: Cost-effectiveness acceptability curve
DANS: Data Archiving and Networked Services
DSM: Diagnostic and Statistical Manual of Mental Disorders
EQ-5D-5L: EuroQoL-5, including the visual analogue scale, 5 items
GEE: Generalized estimating equation
GRP: Good Research Practice research certification
IDS-SR: Inventory of Depressive Symptomatology, Self-Report, 30-items
IMR: Illness Management and Recovery
ISPOR: International Society for Pharmacoeconomics and Outcomes Research-Task Force principles
LUMC: Leiden University Medical Center
MDD: Major depressive disorder
MINI: Mini-International Neuropsychiatric Interview-Dutch translation
NZA: Dutch Healthcare Authority (Nederlandse Zorgautoriteit)
PDD: Persistent depressive disorder
PPEP: Patient and Partner Education Program
PPEP4All: Patient and Partner Education Program for All Chronic Illnesses
QALYs: Quality-adjusted life years
ROM: Routine Outcome Monitoring
SemCAD: Self-Management For Chronic Anxiety and Depression
SPIRIT: Standard Protocol Items: Recommendations for Interventional Trials
SQ-48: Symptom Questionnaire-48, 48 items
SRH: Self-Rated Happiness survey, 1 item
TiC-P: Trimbos Medical Technology Assessment questionnaire for psychiatric illness-associated costs, 30 items
WRAP: Wellness Recovery Action Plan
