# Supplementary material for: The clinical and cost-effectiveness of a self-management intervention for patients with persistent depressive disorder and their partners/caregivers: study protocol of a multicenter pragmatic randomized controlled trial
Source: Trials. 2021 Oct 23;22:731. doi: 10.1186/s13063-021-05666-y (PMC8542316; doi:10.1186/s13063-021-05666-y)
Supplement: Supplementary file 1 — Additional file 1. [file 13063_2021_5666_MOESM1_ESM.doc]

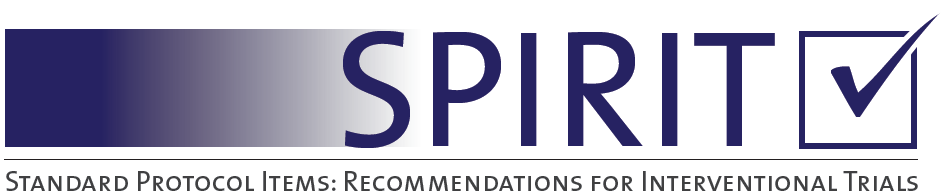


SPIRIT 2013 Checklist: Recommended items to address in a clinical trial protocol and related documents*

| Section/item | ItemNo | Description |
| --- | --- | --- |
| **Administrative information** | | |
| Title | 1 | Descriptive title identifying the study design, population, intervention  Page 1 |
| Trial registration | 2a | Trial identifier and registry name.  Trial registration: NTR5973 on 31 August 2016.  Registry name: Netherlands Trial Register.  <https://www.who.int/ictrp/network/ntr2/en/>  Page 2. |
|  | 2b | World Health Organization Trial Registration Data Set  See <https://www.trialregister.nl/trial/5818> |
| Protocol version | 3 | Date and version identifier  Page 26 |
| Funding | 4 | Sources and types of financial, material, and other support  Page 28 |
| Roles and responsibilities | 5a | Names, affiliations of protocol contributors: page 1, 28  Roles of protocol contributors: page 28 |
|  | 5b | Name and contact information for the trial sponsor: page 15 |
|  | 5c | Role of study sponsor/funder in study design, data collection, analysis, and interpretation of data; writing of the report.  Page 28 |
|  | 5d | Composition, roles, and responsibilities of the coordinating centre, steering committee, endpoint adjudication committee, data management team, and other individuals or groups overseeing the trial, if applicable (see Item 21a for data monitoring committee)  Page 28 |
| Introduction |  |  |
| Background and rationale | 6a | Description of research question and justification for undertaking the trial, including summary of relevant studies  Page 3-4 |
|  | 6b | Explanation for choice of comparators  Page 3-4 |
| Objectives | 7 | Specific objectives or hypotheses  Page 4 |
| Trial design | 8 | Description of trial design including type of trial (RCT), and framework (superiority)  page 4-5, 16 |
| Methods: Participants, interventions, and outcomes | | |
| Study setting | 9 | Description of study setting, study sites  Page 5 |
| Eligibility criteria | 10 | Inclusion and exclusion criteria for participants  Page 5-6  individuals who will perform the intervention  Page 11-12 |
| Interventions | 11a | Interventions for each group with sufficient detail to allow replication, including how and when they will be administered  Page 11-12  Figure 3 |
|  | 11b | Criteria for discontinuing intervention for a given trial participant  page 7, 26 |
|  | 11c | Strategies to improve adherence to intervention protocols, and any procedures for monitoring adherence (treatment integrity)  Page 11, 15-16, 26 |
|  | 11d | Relevant concomitant care and interventions that are permitted or prohibited during the trial  NA- No specific care or interventions prohibited. |
| Outcomes | 12 | Primary, secondary, and other outcomes, including the specific measurement variables analysis metric  Pages 9-11  Figure 2 |
| Participant timeline | 13 | Time schedule of enrolment, interventions (including any run-ins and washouts), assessments, and visits for participants.  Page 6-9  Figure 1 |
| Sample size | 14 | Estimated number of participants needed to achieve study objectives and how it was determined, including clinical and statistical assumptions supporting any sample size calculations  Page 16 |
| Recruitment | 15 | Strategies for achieving adequate participant enrolment to reach target sample size  Pages 25-26 |
| **Methods: Assignment of interventions (for controlled trials)** | | |
| Allocation: |  |  |
| Sequence generation | 16a | Method of generating the allocation sequence (eg, computer-generated random numbers), and list of any factors for stratification.  Page 7 |
| Allocation concealment mechanism | 16b | Mechanism of implementing the allocation sequence (eg, central telephone; sequentially numbered, opaque, sealed envelopes), describing any steps to conceal the sequence until interventions are assigned  Page 22 |
| Implementation | 16c | Who will generate the allocation sequence, who will enrol participants, and who will assign participants to interventions  Page 7 |
| Blinding (masking) | 17a | Who will be blinded after assignment to interventions (eg, trial participants, care providers, outcome assessors, data analysts), and how  Page 4-5, 7 |
|  | 17b | If blinded, circumstances under which unblinding is permissible, and procedure for revealing a participant’s allocated intervention during the trial  Page 22 |
| **Methods: Data collection, management, and analysis** | | |
| Data collection methods | 18a | Plans for assessment and collection of outcome, baseline, and other trial data; page 4-6,9.  description of study instruments along with their reliability and validity;  page 11 |
|  | 18b | Plans to promote participant retention and complete follow-up, including list of any outcome data to be collected for participants who discontinue or deviate from intervention protocols  Page 17 |
| Data management | 19 | Plans for data entry, coding, security, and storage, including any related processes to promote data quality (eg, double data entry; range checks for data values). Reference to where details of data management procedures can be found, if not in the protocol  Page 22 |
| Statistical methods | 20a | Statistical methods for analysing primary and secondary outcomes. Reference to where other details of the statistical analysis plan can be found, if not in the protocol  Pages 16-19 |
|  | 20b | Methods for any additional analyses (eg, subgroup and adjusted analyses)  Page 17, 20-21 |
|  | 20c | Definition of analysis population relating to protocol non-adherence (eg, as randomised analysis), and any statistical methods to handle missing data (eg, multiple imputation)  Page 17 |
| **Methods: Monitoring** | | |
| Data monitoring | 21a | Composition of data monitoring committee (DMC); Alternatively, an explanation of why a DMC is not needed.  Page 22. Not required. |
|  | 21b | Description of any interim analyses and stopping guidelines  Not applicable. |
| Harms | 22 | Plans for collecting, assessing, reporting, and managing solicited and spontaneously reported adverse events and other unintended effects of trial interventions or trial conduct  Page 22 |
| Auditing | 23 | Frequency and procedures for auditing trial conduct, if any, and whether the process will be independent from investigators and the sponsor page 22 |
| Ethics and dissemination | | |
| Research ethics approval | 24 | Ethics approval  Page 22 |
| Protocol amendments | 25 | Plans for communicating important protocol modifications (eg, changes to eligibility criteria, outcomes, analyses) to relevant parties (eg, investigators, REC/IRBs, trial participants, trial registries, journals, regulators)  Page 22 |
| Consent or assent | 26a | Who will obtain informed consent or assent from potential trial participants or authorised surrogates, and how (see Item 32)  Page 6-7, 22 |
|  | 26b | Additional consent provisions for collection and use of participant data and biological specimens in ancillary studies, if applicable  Not applicable. No biological specimens will be taken. |
| Confidentiality | 27 | How personal information about potential and enrolled participants will be collected, shared, and maintained in order to protect confidentiality before, during, and after the trial  Page 22-23 |
| Declaration of interests | 28 | Financial and other competing interests for principal investigators for the overall trial and each study site  Page 29 |
| Access to data | 29 | Statement of who will have access to the final trial dataset, and disclosure of contractual agreements that limit such access for investigators  Page 29-30 |
| Ancillary and post-trial care | 30 | Provisions, if any, for ancillary and post-trial care, and for compensation to those who suffer harm from trial participation  Not applicable, no provisions planned, no major risks associated with study participation |
| Dissemination policy | 31a | Plans for investigators and sponsor to communicate trial results to participants, healthcare professionals, the public, and other relevant groups (eg, via publication, reporting in results databases, or other data sharing arrangements), including any publication restrictions  Page 22-23, 30 |
|  | 31b | Authorship eligibility guidelines and any intended use of professional writers  All named authors adhere to the authorship guidelines of Trials. All authors have agreed to publication.  Page 29-30 |
|  | 31c | Plans, if any, for granting public access to the full protocol, participant-level dataset, and statistical code  Page 23, 29-30 |
| Appendices |  |  |
| Informed consent materials | 32 | Model consent form and other related documentation given to participants and authorised surrogates  The consent form and materials are available from the corresponding author on request. |
| Biological specimens | 33 | Plans for collection, laboratory evaluation, and storage of biological specimens for genetic or molecular analysis in the current trial and for future use in ancillary studies, if applicable  Not applicable. No biological samples will be taken. |
