# Supplementary material for: The clinical and cost-effectiveness of a self-management intervention for patients with persistent depressive disorder and their partners/caregivers: study protocol of a multicenter pragmatic randomized controlled trial
Source: Trials. 2021 Oct 23;22:731. doi: 10.1186/s13063-021-05666-y (PMC8542316; doi:10.1186/s13063-021-05666-y)
Supplement: Supplementary file 2 — Additional file 2. [file 13063_2021_5666_MOESM2_ESM.docx]

**Topic list A**: Patients and partners/caregivers coping with chronic depression

(partly based on topic list in Chambers et al., 2015).

- **Can you tell me what it’s like for you living (*or to live with someone)* with long-term depression?**

*Probe: How long have you experienced depression? What was the cause of the depression?*

- **How has your life changed due to depression (*of the patient)*?**

*Probe: which changes were there in your life?*

*Probe: what made those changes happen?*

*General prompts: daily activities, health-related matters such as medication, diet, alcohol use and/or drugs, body/energy levels, study or work, relationships, religion/spirituality.*

- **What kind of activities can you do less well or not anymore due to the depression *(or due to caring for a person/the patient with depression)*?**
- **What do you do to feel a bit better when you are feeling depressed? *(or how do you try to help a person with depression?)* (Coping)**

*Probe: How do these things help?*

- **Which family members or friends support you the most? *(or are there areas for which you would you like to receive more support?)***

*Probe:* *How do they help or support you?*

*Probe: What could they do to help more?*

- **How do you know if something or someone is helping or not?***

*Probe: how could you experience or see that it is helping you?*

- **When you’re going through a particularly bad time, how do you handle this?***
- **When things are going well, what do you do to keep that going?****Probe: What are helping things for you then?*
- **Some people talk about self-determination, or being in charge of their own life. How in charge of your life are you?**

*Probe:* *What gives you hope in your life?*

- **Are there any other problems that you experience besides depression, for which you would like to receive help or support?**

*General prompts: physical complaints, social problems (e.g., with family/relational problems, friendships), work, problems with daily activities and selfcare (e.g., eating/cooking, doing groceries, cleaning, child care, or care for another).*

- **Is there anything else you would like to add that we may not have asked?**

* These questions are not included in the interview topic list for the partner/caregiver.

*General prompts that can be used at any time during the interview:*

- What was that like for you?
- Can you tell me more about that?
- And then what happened?
- Is there anything else you’d like to add?
- Have you mentioned everything you’d like to?
