# Supplementary material for: The clinical and cost-effectiveness of a self-management intervention for patients with persistent depressive disorder and their partners/caregivers: study protocol of a multicenter pragmatic randomized controlled trial
Source: Trials. 2021 Oct 23;22:731. doi: 10.1186/s13063-021-05666-y (PMC8542316; doi:10.1186/s13063-021-05666-y)
Supplement: Supplementary file 3 — Additional file 3. [file 13063_2021_5666_MOESM3_ESM.docx]

**Topic list B.** Patient, partner/caregiver, and therapist satisfaction with PPEP4All
(partly based on Mental Healthcare Thermometer (Kok & van Wijngaarden, 2003)).

- **How was your experience with PPEP4All?**
  *Probe: what did you think about it?*
- **What effect/outcome did PPEP4All have on you?***
- **What did you learn from PPEP4All?***
- **Did you make any changes in your life due to PPEP4All?***
  *Probe: Would your life be any different if you had not followed this program?*
- **What did you think about your PPEP4All-therapist?***
- **What did you like about PPEP4All?**
  *Probe: what were the positive points?*
- **What didn’t you like about PPEP4All?**
  *Probe: what were the negative or less positive points?*
- **How would you rate PPEP4All?** [where 1 = awful and 10 = outstanding]
  *Probe: could you elaborate why you gave this score?*
- **Do you have any other comments or suggestions to further improve/optimize PPEP4All?**

* These questions were not included in the interview topic list for PPEP4All-therapists.

*General prompts that can be used at any time during the interview:*

- Can you tell me more about that?
- Is there anything else you’d like to add?
- Have you mentioned everything you’d like to?

*Specific prompts regarding PPEP4All, ask more about:*

- the themes and/or information of the program;
- homework and/or activities;
- the participant workbook;
- the therapist handbook (only for PPEP4All-therapists);
- treatment location.
